# The relationship between psychological capital, burnout and perceived stress in junior nurses: a latent profile analysis

**DOI:** 10.3389/fpubh.2024.1374941

**Published:** 2024-04-10

**Authors:** Xu Zhang, Siye Chen, Ziling Zheng, Mi Zhao, Li Song, Yue Zhao, Zhiwen Wang

**Affiliations:** ^1^School of Nursing, Peking University, Beijing, China; ^2^Department of Nursing, Peking University First Hospital, Beijing, China

**Keywords:** junior nurses, psychological capital, burnout, perceived stress, latent profile analysis

## Abstract

**Background:**

Psychological capital, an intrinsic personal asset, enhances junior nurses’ ability to navigate transition and sustain superior job performance. This study aimed to classify junior nurses into distinct psychological capital profiles and examine their associations with burnout and perceived stress levels.

**Methods:**

A cross-sectional study involving 480 junior nurses from three hospitals in Beijing assessed psychological capital, stress, and burnout using e-questionnaires, from July 2021 to August 2022. We employed exploratory latent profile analysis for psychological capital profiling and logistic regression with the best subset method to identify the influential factors.

**Results:**

The results of the latent profile analysis supported the models of two latent profiles, which were defined as low psychological capital (224, 46.5%) and high psychological capital (256, 53.5%). Logistic regression revealed that introverted nurses and those experiencing moderate to high levels of burnout and stress were more likely to exhibit low psychological capital.

**Conclusion:**

Nursing management should proactively identify and support junior nurses with low psychological capital, with a focus on introverted individuals, to mitigate the impact of stress and burnout.

## 1 Background

With the development of positive psychology and active organizational behavior, researchers have gradually shifted their focus from negative psychology to positive mental states and the behavioral attitudes of individuals. Initially, Luthans introduced psychological capital to explain the positive mental abilities of employees (1), which refers to a type of positive mental energy expressed in the growth and development of an individual, indicating an individual’s motivational tendencies, and is a higher-order composite multidimensional structure generated through positive mental structures. Previous studies have demonstrated that nurses’ psychological capital is positively correlated with desired work-related outcomes such as work engagement, job satisfaction, organizational commitment, and innovative behaviors (2). Higher psychological capital was also associated with lower undesirable work-related outcomes like burnout and turnover intentions. (3, 4). In addition, psychological capital promotes nurses’ mental health and relieves work stress; for example, previous research has confirmed that psychological capital improves depression and insomnia among nurses (5–7). Based on the above, in today’s global background of nursing human resource shortage, improving nurses’ psychological capital can help to further improve their physical and mental health, stabilize and develop the nursing workforce, and improve the performance of organizational management (8). Despite the benefits of psychological capital, a meta-analysis showed a downward trend in nurses’ psychological capital levels starting in 2019 (9). In addition, it is worth noting that among the group of nurses, junior nurses, who are the mainstay of future nursing human resources, but have the lowest level of psychological capital, deserve more attention (10).

Nurses who have worked in the clinical setting for less than 3 years after graduating from the nursing profession are called junior nurses (11). By 2025, more than one million registered nurses are expected to retire, and these vacancies will be filled by junior nurses (12). The WHO reported on the State of the World’s Nursing 2020 also notes that the nursing workforce is relatively young (13). As an important human resource pool for the nursing team, junior nurses are relevant to the continued health of the future nursing workforce (14). A scoping review noted that nurses with younger age and years of experience possess lower psychological capital, which is detrimental to the development of optimistic work attitudes and clinical thinking skills, reduces work satisfaction and adaptability, and contributes to increased turnover rates among junior nurses (15, 16). In 2023, registered nurses leaving within 2 years of employment account for 52.5% of all departing nurses, and the cost per registered nurse leaving a job average about $52,350 (17). The reason for such a high turnover rate may be that junior nurses have just gone through the transition from school to hospital, and are faced with the impact of the gap between theory and practice, which are vulnerable to maladaptation because of their lack of knowledge and work experience and their confusion about their future development (18, 19). It has been shown that newly graduated registered nurses are particularly vulnerable to emotional exhaustion, stress and burnout during their first 3 years on the job (20). Psychological capital, as an internal personal resource, can help junior nurses cope with transitions and effectively manage work stress (21, 22). Therefore, there is a need to focus on nurses’ psychological capital and the factors that influence it in order to identify nurses with low psychological capital and lay the foundation for subsequent support and intervention.

There are no cut-off scores that distinguish between different levels of psychological capital and the complexity of factors influencing psychological capital may be subject to heterogeneity between groups. Most existing studies have adopted a variable-centered approach, which is limited to exploring the correlation between variables and ignoring the existence of heterogeneity among individuals. Thus, research on the differences in psychological capital of nurses with different characteristics is lacking, which is not conducive to identifying people with different characteristics and proposing targeted interventions accordingly (23). Furthermore, there are still no standardized criteria for categorizing psychological capital, and classifying it based only on the scores of the scales ignores the differences between individuals. However, individual-centered latent profiling analysis (LPA) avoids these problems. LPA is a person-centered approach that allows individuals with similar personal and professional characteristics, traits, or behavioral patterns to be combined into profiles based on their responses and has been applied to explore nurses’ work engagement, moral courage, professional benefit, and burnout (24–27). Through LPA, different profiles of psychological capital of junior nurses were identified, and subgroups with heterogeneous characteristics and differences in influencing factors between groups were explored, thus providing new perspectives on targeted measures to prevent perceived stress, burnout, and psychological outcomes for nurses, improve their clinical performance, and reduce turnover rates.

Therefore, this study used LPA to explore the potential profiles of psychological capital of junior nurses from an “individual-centered” perspective and conducted an in-depth quantitative analysis of the factors affecting the potential profiles of psychological capital, with a view to providing a reference for the development of scientific and effective individualized interventions for the psychological capital of junior nurses to promote high-quality and sustainable development of the nursing workforce.

## 2 Materials and methods

### 2.1 Aims

This study aimed to explore subsets of psychological capital among junior nurses using person-centered latent profile analysis and to identify the factors associated with a certain psychological capital profile.

### 2.2 Design

This was a multicenter cross-sectional investigation. The study followed the Strengthening the Reporting of Observational Studies in Epidemiology (STROBE) guidelines (28).

### 2.3 Study participants and sample size

This study used an online recruitment method with junior nurses from three grade A tertiary hospitals in Beijing, China. The survey was conducted from July 2021 to August 2022. The study participants were required to meet the following criteria: (1) being 18 years of age or older, (2) having a maximum of 3 years of work experience, and (3) providing informed consent. To ensure the validity of the results, nurses with psychological diseases or those on a long leave of absence were excluded from the study.

The PASS2021 software was used for the sample size calculation. Based on a systematic evaluation of the level of nurses’ psychological capital published in 2023 (9), we calculated the standard deviation of Asian nurses’ psychological capital scores to be approximately 31.18. Setting α = 0.05, the distance from the mean to the limit was 3.00, and the required sample size was calculated to be 418 (29). Considering the incorrectly filled in or non-response, the sample size was further augmented by 10%, which ultimately determined that the sample size to be surveyed for this study was 465.

### 2.4 Measures

#### 2.4.1 Baseline characteristics of the participants

Designed by the research team, it collected the individual characteristics of the participants and work-related factors. The individual characteristics of the participants included age, gender, education level, salary income, and personality. Work-related factors included doctor-nurse relationships, night shifts, and turnover intention.

#### 2.4.2 Psychological capital questionnaire (PCQ)

The scale was developed by Luthans et al. to measure the psychological capital of research participants (30). Luo et al. translated and revised the scale into a Chinese version through cross-cultural debugging in 2010 (31). The revised Chinese version of the scale has 20 items, including four dimensions: self-efficacy (1–6 items), hope (7–12 items), resilience (13–17 items), and optimism (18–20 items). A 6-point Likert scale was used, with a total score ranging from 20 to 120, with higher scores indicating higher levels of psychological capital. The McDonald’s ω of the scale was 0.835 in this study.

#### 2.4.3 Maslach burnout inventory (MBI)

The scale was developed by Maslach et al. to measure burnout among medical professionals (32). The Chinese version translated by Feng et al. was used in this study (33). It consists of 22 items and includes three dimensions: emotional exhaustion, depersonalization, and personal accomplishment. The scale is based on a 7-point Likert scale with a total score of 0–132, with higher scores indicating more severe burnout. To facilitate interpretation of the results, the dimension of personal achievement was reverse-graded in this study and named low personal accomplishment. The McDonald’s ω of the scale was 0.770 in this study.

#### 2.4.4 Perceived stress scale (PSS)

The scale was developed by Cohen et al. to measure the degree of perceived psychological stress in individuals and translated into Chinese by Yang (34, 35). The Chinese version of the PSS has a total of 14 entries and uses a 5-point Likert scale, with a total score ranging from 0 to 56. A score greater than 24 is considered to be perceived stress, with higher scores indicating greater perceived stress. The McDonald’s ω of the scale was 0.725 in this study.

### 2.5 Data collection

The study team first established collaboration with the department of nursing at each study center, and the initial screening of potential study subjects who met the inclusion and exclusion criteria was conducted by reviewing the induction and attendance records of nurses in each hospital. Administrators of the Department of Nursing at each hospital were commissioned to send an invitation for study recruitment and an informed consent form to potential study participants. An electronic questionnaire was sent through the WeChat platform to nurses who volunteered to participate and signed an informed consent form. To avoid missing data, we set each question of the e-questionnaire as a mandatory field that could only be submitted if all answers were completed. All completed questionnaires were transmitted to the back-end management platform via the Internet to be checked by members of the research team, and participants were asked to refill in any questionnaires that were incorrectly filled in or clearly inconsistent with the actual situation.

### 2.6 Ethical consideration

The study adhered to the principles set forth in the Declaration of Helsinki and was thoroughly examined and endorsed by the Medical Ethics Committee of Peking University First Hospital (No: 2021–415). Prior to their inclusion in the study, all individuals willingly provided informed consent, and stringent measures were implemented to ensure the anonymity of all recorded and analyzed data.

### 2.7 Statistical analyzes

Data were statistically analyzed in this study using Mplus 8.3 and R software (version 4.3.1), and a two-sided test of *p* < 0.05 indicated a statistically significant difference. We used frequency/percentage (%) for the statistical description of count or rank information in participants’ baseline data; continuous variables such as participants’ age and scale scores were first plotted on Q-Q plots to test the normality of the data. The majority of the points of the above variables could be distributed on a straight line with a clear linear trend, so the continuous data could be considered to follow a normal distribution and were therefore described using Mean ± SD. Pearson’s correlation was used to analyze the correlation coefficients between the total scores and dimensions of each scale.

An exploratory latent profile analysis was used to explore latent models of psychological capital in junior nurses. We fitted a total 1–5 profile models, after which the best model was screened by comparing each model index. The model indices used in this study included the Pearson chi-square test, likelihood ratio chi-square test, Akaike information criterion (AIC), Bayesian information criterion (BIC), and sample-corrected BIC (aBIC), to identify the goodness of fit of the model, with smaller values representing a better fit. The bootstrap likelihood ratio test (BLRT) and lo–mendell–rubin (LMR) were used to compare the differences in fit between the models, and *p*-values of both LMR and BLRT that reached a significant level indicated that the model with k categories was significantly better than the model with k-1 categories (36). An entropy value closer to 1 indicates a more accurate classification (37).

Finally, this study constructed a logistic regression model to analyze the effects of the above variables on psychological capital profiles, using the psychological capital profile category as the dependent variable and baseline information, burnout, and perceived stress of junior nurses as independent variables. In the process of model construction, the variance inflation factor was first used to test the multicollinearity of the model, and a VIF < 5 indicated that there was no multicollinearity problem in the model (38). Subsequently, all possible combinations of features were traversed through the best subset method to increase the accuracy of the analysis results, and the features affecting different profiles of psychological capital were determined (39).

## 3 Results

### 3.1 Participant characteristics

A total of 480 junior nurses completed the questionnaire, meeting the sample size requirements for this study. The age of the 480 participants was 24.2 ± 1.88 years, of which 88% were female and 12% were male. The number of those who received a bachelor’s degree or a higher level of education was 296 (62%). When asked about an individual’s personality, 48% gave feedback that they were extroverted, while 52% were introverted. The overall psychological capital score was 80.2 ± 12.84.75% reported moderate-to-high levels of burnout. Additionally, 56% of the participants were stressed. Table 1 presents information on the participants’ general characteristics.

**Table 1:** Patient demographics and baseline characteristics.

Characteristic	*N* = 480^a^
*Age*	
Mean (SD)	24.2 (1.88)
*Gender*	
Female	420 (88%)
Male	60 (12%)
*Education*	
Junior college	184 (38%)
Undergraduate	247 (52%)
Postgraduate	49 (10%)
*Monthly Salary (¥)*	
5,000–10,000	156 (33%)
<5,000	280 (58%)
>10,000	44 (9%)
*Work experience*	
1 year	203 (42%)
2 year	164 (34%)
3 year	113 (24%)
*Personality*	
Extraversion	229 (48%)
Introverted	251 (52%)
*Relationship between doctors and nurses*	
Good	370 (77%)
Poor	110 (23%)
*Rotating night shift work*	
Yes	392 (82%)
No	88 (18%)
*Turnover intention*	
Yes	53 (11%)
No	427 (89%)
*PCQ scores*	
Mean (SD)	80.2 (12.84)
*MBI scores*	
Mean (SD)	76.8 (11.55)
*PSS scores*	
Mean (SD)	25.5 (6.45)
*MBI category*	
Low	122 (25%)
Medium	247 (52%)
High	111 (23%)
*PSS category*	
Normal	209 (44%)
Stressful	271 (56%)

a *n* (%); SD, standard deviation; PCQ, Psychological Capital Questionnaire; MBI, Maslach Burnout Inventory; PSS, Perceived stress scale.

### 3.2 Correlation analysis

**Figure 1:**
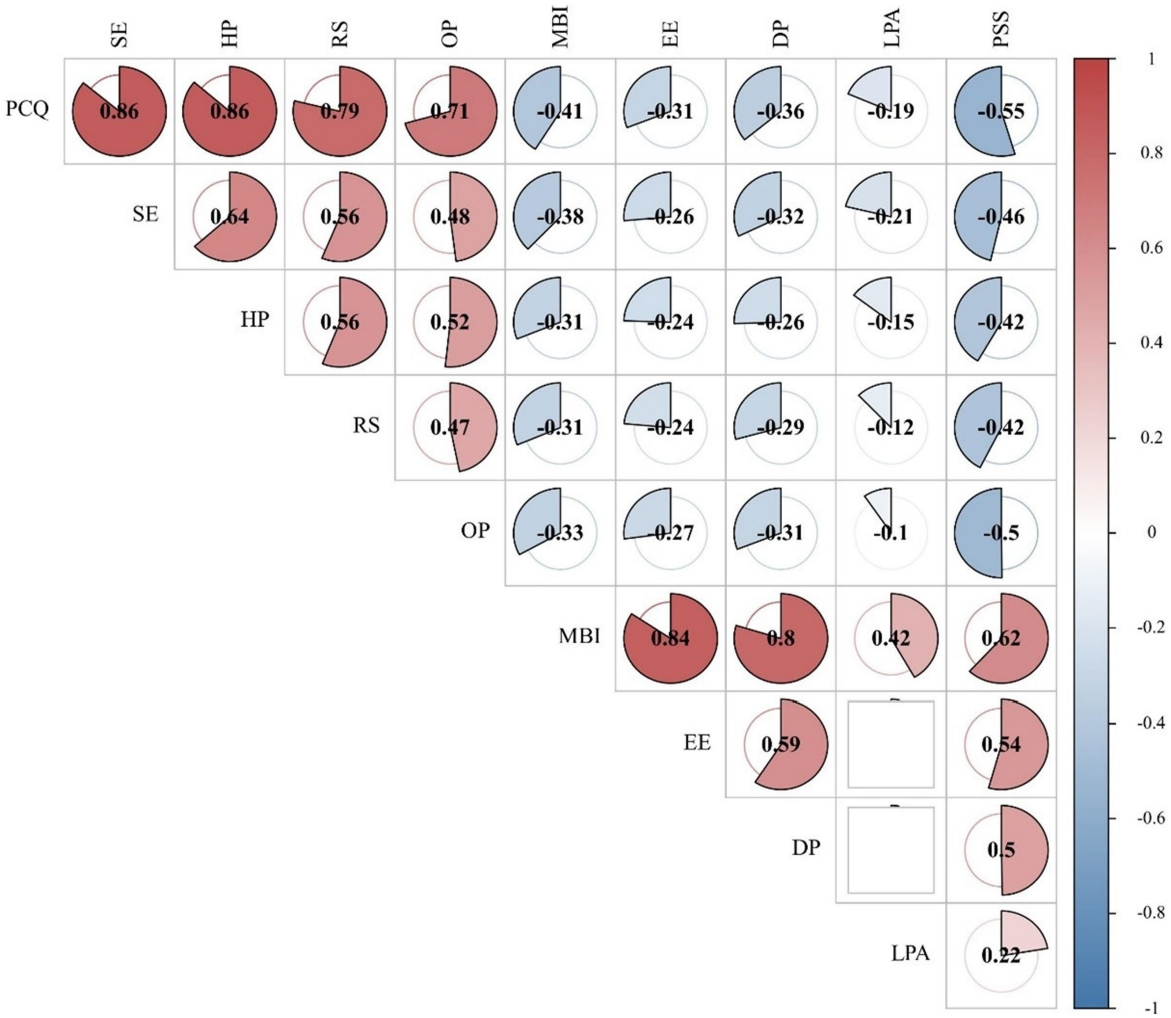
Correlations between psychological capital, burnout, and perceived stress in junior nurses. No statistical significance for white spaces. PCQ, Psychological Capital Questionnaire; SE, Self-Efficacy; OP, Optimism; HP, Hope; RS, Resilience; MBI, Maslach Burnout Inventory; EE, Emotional Exhaustion; LPA, Low Personal Accomplishment; DP, Depersonalization; PSS, Perceived stress scale.

The results of Pearson’s correlation analyses indicated that there were low and medium levels of negative correlations between the total psychological capital scores and each of its dimensions and burnout (including all dimensions) and perceived stress among junior nurses (*r* = −0.1 to-0.55). However, there was a positive correlation between burnout and its dimensions, and perceived stress at medium and high levels (*r* = 0.22 to 0.62). All the results were statistically significant (*p* < 0.05). Figure 1 shows the correlation coefficient plot between psychological capital, burnout, and perceived stress among the junior nurses.

### 3.3 Latent profile analysis of psychological capital

This study conducted an individual-centred latent profile analysis of the psychological capital of 480 junior nurses. Four-dimensional scores of the psychological capital questionnaire were used as exogenous indicators, and a latent profile model ranging from 1 to 5 was obtained by gradually and sequentially fitting the baseline model from latent profile 1. Table 2 shows the model fit information. Although the 5-profile model had the lowest values of AIC, BIC, and aBIC and significant LMR and BLRT. However, the two groups in this model had participant proportions below 0.1% and the results were less interpretable. Among the other latent profiles, the 2-profile model had the largest entropy value above 0.8, indicating the highest classification accuracy and relatively good posterior probability. In addition, Supplementary Figure S1 shows a plot of the information criteria by the number of profiles. Profile 2 had the most pronounced slope of decline in fit, and from there, the improvement in each fit metric leveled off significantly. Therefore, the 2-profile model was selected as the final solution for this study.

**Table 2:** Fit indices for five models using latent profile analysis (*N* = 480).

Latent profile	AIC	BIC	aBIC	LMR(P)	BLRT(p)	Entropy	Group size for each profile	Proportion of each profile
1-profile	10651.45	10684.88	10659.49				480	
**2-profile**	**10109.84**	**10164.15**	**10122.89**	**<0.001**	**<0.001**	**0.811**	**224/256**	**0.465/0.535**
3-profile	10024.87	10100.07	10042.94	0.0052	<0.001	0.793	50/219/211	0.108/0.454/0.438
4-profile	9997.552	10093.65	10020.65	0.0423	<0.001	0.791	195/49/24/212	0.407/0.101/0.050/0.442
5-profile	9955.485	10072.47	9983.598	<0.001	<0.001	0.809	2/56/190/206/26	0.005/0.116/0.398/0.427/0.054

Numbers in bold indicate “best” fit. AIC, Akaike Information Criterion; BIC, Bayesian Information Criterion; aBIC, adjusted BIC; pLMR, *p*-value for Lo–Mendell–Rubin adjusted likelihood ratio test for K vs. K-1 profiles; pBLRT, *p*-value for Bootstrapped Likelihood Ratio Test.

**Figure 2:**
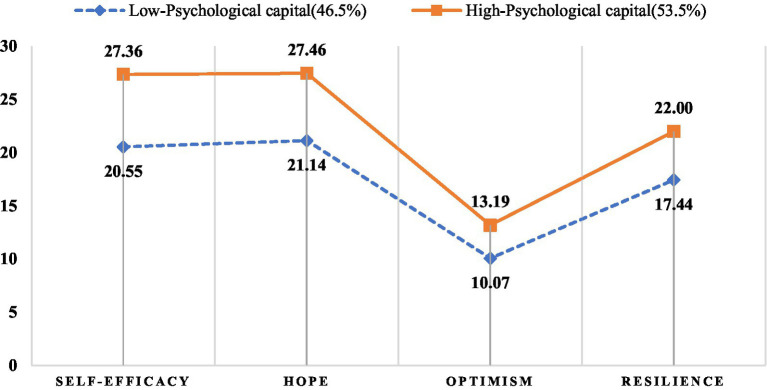
Two-profile model in the present study.

According to the 2-profile results, 224 (46.5%) of the junior nurses belonged to Category 1 and 256 (53.5%) belonged to Category 2. Figure 2 plots the line graphs of the scores on the dimensions of psychological capital to facilitate the analysis of the characteristics of the two profiles of psychological capital of junior nurses. As shown in Figure 2, the fluctuations in the ratings of the dimensions of psychological capital of the two categories of junior nurses were broadly similar, but Category 1 was generally at a lower level than Category 2. The total psychological capital scores for categories 1 and 2 were 68.83 ± 7.59 and 90.14 ± 6.80, respectively. Therefore, this study labelled Category 1 as low psychological capital and Category 2 as high psychological capital.

### 3.4 Factors associated with belonging to a certain psychological capital profile

Supplementary Figure S2 exhibits the VIF values of all independent variables. The VIF values of the variables in this study were all less than five, indicating that there was no multicollinearity. The best subset method was used to screen the associated factors belonging to a certain psychological capital profile using the multiple logistic regression model (Supplementary Figures S3, S4). The findings suggest that self-reported introverted individuals are more likely to belong to the low psychological capital category than extroverted junior nurses (OR = 0.65, 95% CI: 0.43–0.98); junior nurses at moderate and high burnout levels were more likely to be in the low psychological capital category compared to low burnout (OR = 0.57, 95% CI: 0.33–0.97; OR = 0.30, 95% CI: 0.15–0.59); nurses who perceived stress were also likely to be in the low psychological capital category (OR = 0.18, 95% CI: 0.11–0.29). Detailed results are presented in Table 3.

**Table 3:** Factors associated with belonging to a certain psychological capital profile (Logistic regression).

	Univariable					Multivariable		
Characteristic	*N*	Event N	OR^a^	95% CI^a^	*p*-value	OR^a^	95% CI^a^	*p*-value
*Age*	480	256	1.04	0.95, 1.15	0.409			
*Gender*								
Female	420	223	—	—				
Male	60	33	1.08	0.63, 1.87	0.782			
*Personality*								
Extraversion	229	135	—	—		—	—	
Introverted	251	121	0.65	0.45, 0.93	0.019	0.65	0.43, 0.98	0.041
*Education*								
Junior college	184	105	—	—				
Undergraduate	247	122	0.73	0.50, 1.08	0.115			
Postgraduate	49	29	1.09	0.58, 2.09	0.790			
*Work experience*								
1 year	203	90	—	—				
2 year	164	87	1.42	0.94, 2.15	0.097			
3 year	113	79	2.92	1.80, 4.79	<0.001			
*Monthly Salary (¥)*								
5,000–10,000	156	82	—	—				
<5,000	280	150	1.04	0.70, 1.54	0.840			
>10,000	44	24	1.08	0.55, 2.13	0.816			
*Rotating night shift work*								
Yes	392	206	—	—				
No	88	50	1.19	0.75, 1.90	0.469			
*Relationship between doctors and nurses*								
Good	370	211	—	—				
Poor	110	45	0.52	0.34, 0.80	0.003			
*Turnover intention*								
Yes	53	20	—	—				
No	427	236	2.04	1.14, 3.72	0.017			
*MBI category*								
Low	122	94	—	—		—	—	
Medium	247	133	0.35	0.21, 0.56	<0.001	0.57	0.33, 0.97	0.041
High	111	29	0.11	0.06, 0.19	<0.001	0.30	0.15, 0.59	<0.001
*PSS category*								
Normal	209	166	—	—		—	—	
Stressful	271	90	0.13	0.08, 0.19	<0.001	0.18	0.11, 0.29	<0.001

Reference, low psychological capital.

a OR, Odds Ratio, CI, Confidence Interval Number in dataframe = 480, Number in model = 480, AIC = 549.5, C-statistic = 0.777, H&L = 2.18 (*p* = 0.975).

## 4 Discussion

The results of a meta-analysis showed that Asian nurses had lower levels of psychological capital than those in the Americas and Australia, which may be related to increased work stress and negative workplace experiences due to the shortage of human resources for nursing (9, 40). Therefore, more attention should be paid to the psychological capital of Asian nurses and interventions should be implemented. This study was the first to use LPA to categorize the psychological capital of junior nurses and to explore the factors that influence the level of psychological capital in different categories.

The study found that the psychological capital of junior nurses is at a moderate-high level, but still lower than the average for nurses (9) and the results of a previous study of junior nurses (21). This suggests that the psychological capital of junior nurses deserves attention. As the mainstay of the future nursing workforce, junior nurses should be prepared by their managers to take effective measures to prepare them for the transition from school to clinic (41). Lower than the study (21) may be due to the time of year the study was conducted and the differences between geographical areas. The time of data collection in this study was during COVID-19, and nurses faced the unpredictable development of COVID-19, decreased confidence in coping with and overcoming the outbreak, feared contracting the virus on the job and bringing it back to their families; they were skeptical of their ability to competently care for patients with COVID-19, possessed lower optimism and self-efficacy, and consequently, had lower psychological capital (42). This study used latent profiling analysis to identify two potential categories of psychological capital of junior nurses, namely the low-psychological capital group and the high-psychological capital group, with significant differences in scores between the two groups, and the number of people in each group was almost equal to half; therefore, managers should identify junior nurses with different levels of psychological capital before adopting more targeted and effective interventions to avoid wasting resources. On one hand, the focus is on the low psychological capital group of junior nurses, implementing psychological interventions to improve their psychological capital level. On the other hand, the aim is to sustain the psychological capital level of junior nurses in the high psychological capital group, ensuring they can pass through the early stage of their working life healthily and smoothly, and maintain this positive psychological resource in the long-term future of their working life.

Compared to extroverted junior nurses, nurses who self-reported as introverted had a greater probability of belonging to the low psychological capital category. A study of mental health workers also found that extroverted personality was positively correlated with psychological capital, meaning that extroverted people were more likely to have higher psychological capital (43). The reason may be that extroverts have better socialization, are active and enthusiastic, easily establish harmonious and stable relationships and effective communication with patients and colleagues at work, and obtain more social support (44). Social support, as an important external support for junior nurses, can enhance their self-confidence and expectation of future success, and also help junior nurses to strengthen their beliefs, positively cope with difficulties and challenges, and improve their resilience in the face of setbacks (45). At the same time, extroverts are usually more confident and optimistic and thus have higher levels of psychological capital.

The results of this study indicate that junior nurses who perceive stress are likely to belong to the low psychological capital category. Although every nurse faces a variety of stressors in their work scenario, the impact of these stressful events depends in part on how the individual perceives the stress generated by these events to be (46, 47). Junior nurses with low psychological capital are accustomed to characterizing stressful events with negative perceptions, thus perceiving more stress and being affected by it (48, 49). On the other hand, if junior nurses always perceive too much stress in their work, they may develop negative thinking, which in turn can reduce psychological capital (5). Therefore, in cases where some stressful events in the work process of junior nurses cannot be avoided, negative effects such as negative emotions, burnout, and other negative effects of perceived stress and stress can be reduced by improving the psychological capital of junior nurses (47). It has been shown that participation in psychological training is a protective factor for nurses’ psychological capital, so managers can carry out mental health courses or lectures for junior nurses, such as positive stress reduction training, resilience training, and other more mature psychological interventions, in order to reduce the level of stress and improve the ability to withstand the face of setbacks, thus improving the level of psychological capital (50).

The results of this study indicated that the higher the burnout, the higher the probability that a junior nurse would be classified in the Low-psychological capital category, similar to the results of previous studies (51). The four dimensions of self-efficacy, hope, optimism, and resilience contained in psychological capital are themselves burnout antagonists, reducing emotional exhaustion and inhibiting burnout, so psychological capital as a positive psychological resource directly affects burnout (52, 53). Furthermore, psychological capital indirectly affects burnout by influencing nurses’ assessment and coping styles in response to stressful events (5). Junior nurses are confronted with more stressful events in the workplace due to their inexperience in the workplace, and junior nurses with high levels of psychological capital are more likely to assess such stress as normal pressures and challenges rather than burdens and injuries, and to adopt active coping styles to solve problems and enhance their competence (54). Positive emotions and behaviors that accompany this process lead to lower levels of emotional exhaustion and decreased personal accomplishment, thus avoiding burnout (55). A Korean study on burnout among clinical nurses also suggested that positive emotions were the strongest explanatory factor for burnout (51). From an organizational perspective, organizational commitment mediates the relationship between psychological capital and burnout; nurses with greater psychological capital are more willing to contribute to the organization and identify more strongly with the team and the various types of support they receive from the organization, resulting in higher levels of occupational well-being and lower levels of emotional exhaustion and cynicism, and lower levels of burnout (56). Previous studies have pointed out that the four dimensions of psychological capital can be approached to achieve the goal of improving psychological capital, and the researcher has developed a framework to improve HERO with specific measures including conducting “hope huddles” with teammates, practicing positive self-talk, reflecting on past successes and so on (57). Meanwhile, reliable lead teachers can be set up for junior nurses to provide them with supportive guidance and role modeling, to jointly set work goals, to discuss confusions encountered in their work, and to enhance positive emotions and professionalism to promote the improvement of psychological capital (58).

This study also has some limitations, such as the cross-sectional design of this study, which makes it difficult to explore the causal relationship between variables and needs to be followed up with a longitudinal study. The study was conducted only in Beijing, China, which is not representative of individuals from other countries or cultures, and a multicenter, large-sample study could be carried out in the future. The use of a convenience sampling method may lead to selection bias and the overrepresentation of certain groups, which restricts the results of the extrapolation.

### 4.1 Implications for nursing management

Psychological capital, as a positive psychological resource, has a favorable impact on nurses (59). In the context of the global shortage of nurses, junior nurses, as the mainstay of the future nursing workforce, experience role transitions and are prone to maladjustment and a variety of psychological problems that have not received the attention they deserve (60). This study provides managers with information about the psychological capital of junior nurses, explores the relationship between different categories of psychological capital and adverse outcomes, and suggests that managers should identify nurses with low psychological capital and target interventions, including special attention to introverted nurses, among others, to avoid the effects of high perceived stress and burnout.

## 5 Conclusion

The psychological capital of junior nurses is in the moderate to high level, but still lower than that of the nurses’ group. The latent profile analysis confirms the existence of obvious classification characteristics of psychological capital of junior nurses, which not only provides a basis for classifying the level of psychological capital, but also suggests that managers should identify junior nurses with low psychological capital in a timely manner and take targeted interventions to reduce the level of perceived stress and burnout, to help junior nurses adapt to clinical work as soon as possible, and to maintain the stability and sustainable development of the nursing workforce.

## Data availability statement

The raw data supporting the conclusions of this article will be made available by the authors, without undue reservation.

## Ethics statement

The studies involving humans were approved by Medical Ethics Committee of Peking University First Hospital (No: 2021–415). The participants provided their written informed consent to participate in this study.

## Author contributions

XZ: Conceptualization, Formal analysis, Methodology, Software, Validation, Visualization, Writing – original draft. SC: Conceptualization, Formal analysis, Methodology, Software, Validation, Visualization, Writing – original draft. ZZ: Data curation, Investigation, Resources, Writing – original draft. MZ: Data curation, Investigation, Resources, Writing – original draft. LS: Data curation, Investigation, Resources, Writing – original draft. YZ: Conceptualization, Funding acquisition, Project administration, Supervision, Writing – review & editing. ZW: Conceptualization, Project administration, Supervision, Writing – review & editing.
