# Supplementary material for: The relationship between psychological capital, burnout and perceived stress in junior nurses: a latent profile analysis
Source: Front Public Health. 2024 Apr 10;12:1374941. doi: 10.3389/fpubh.2024.1374941 (PMC11039913; doi:10.3389/fpubh.2024.1374941)

| FigureS1 Five Model Parameter Variations |
| --- |

FigureS2 Variance inflation factor (VIF) of the variables in the model


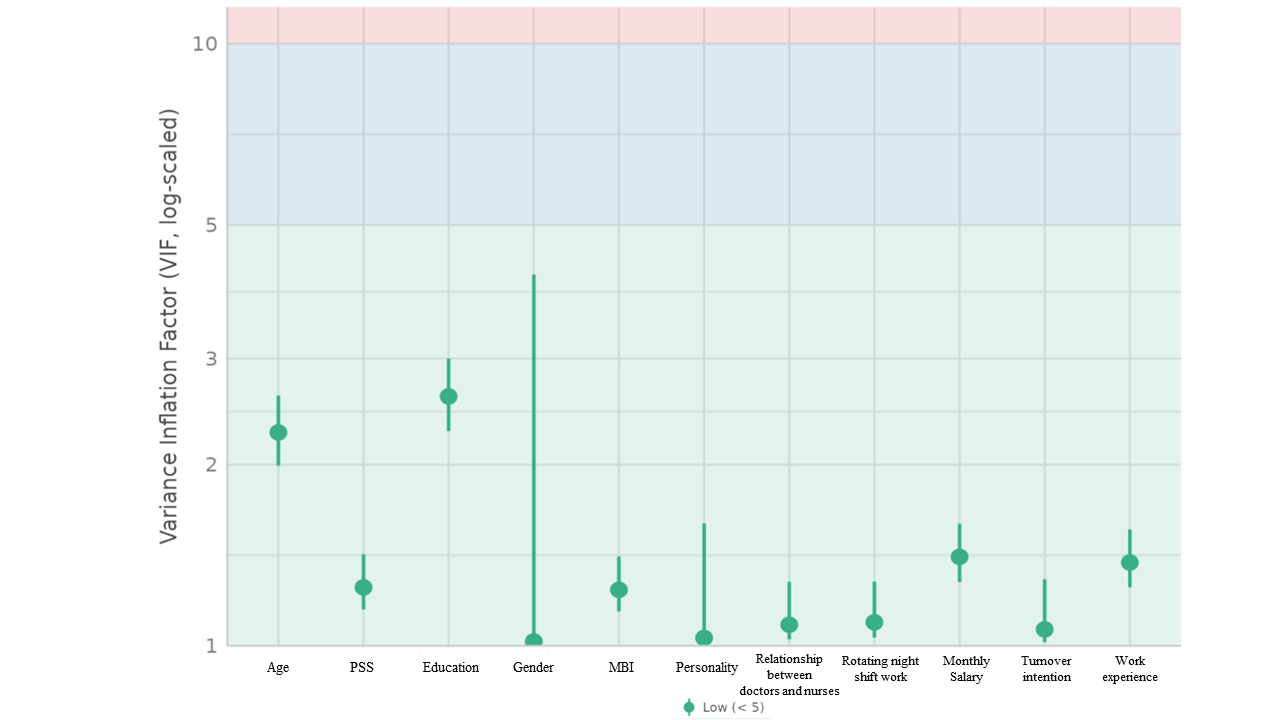


FigureS3 Filtering Variables Using Best Subsets


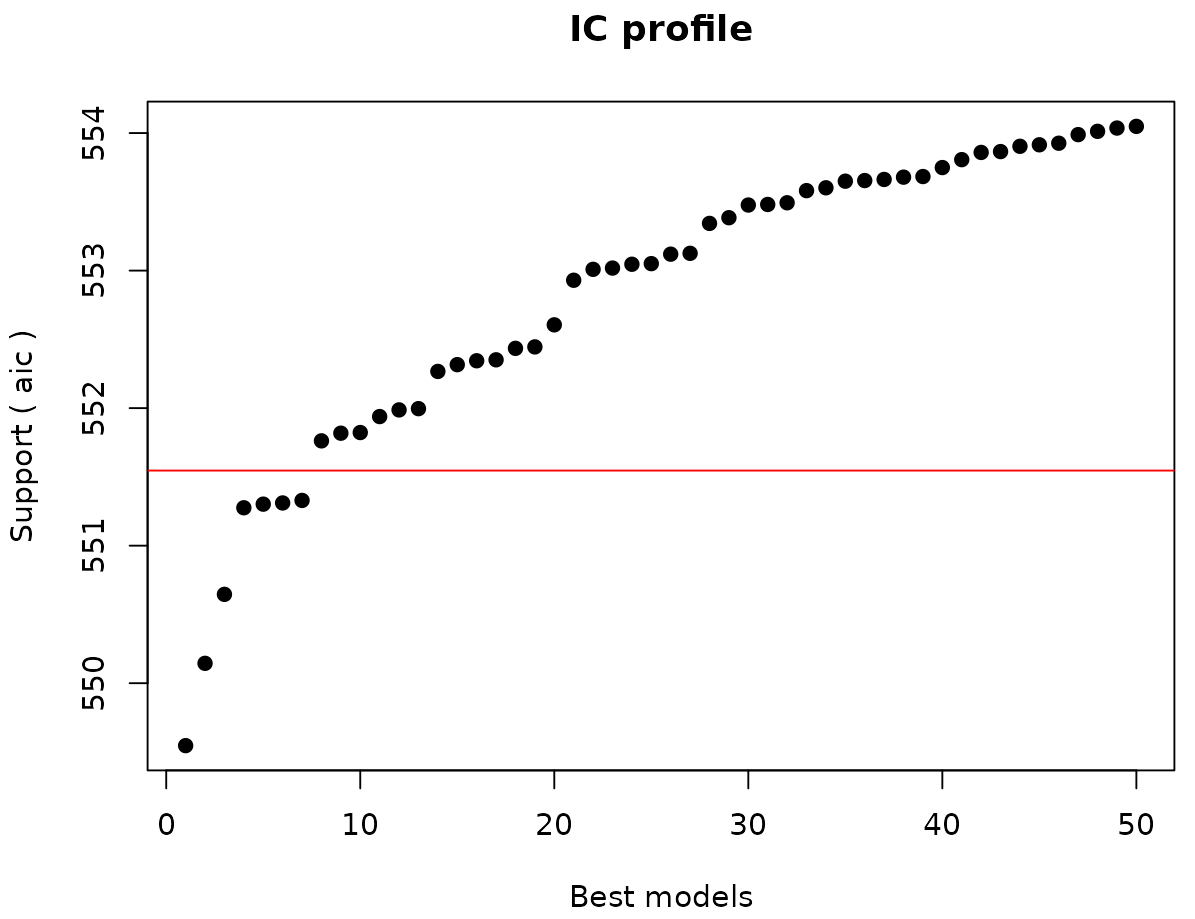


FigureS4 Average importance of the variables in the model


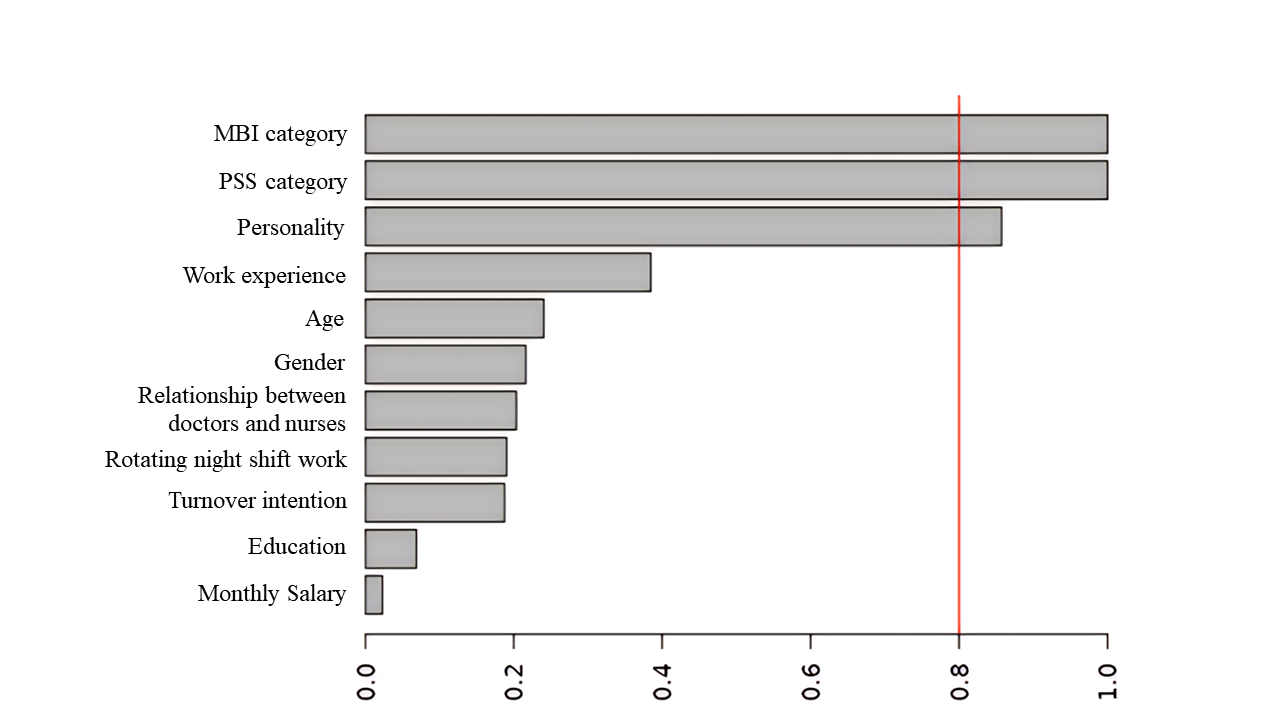

Supplement: Supplementary file 1 [file Data_Sheet_1.docx]
